# Modeling and Density Estimation of an Urban Freeway Network Based on Dynamic Graph Hybrid Automata

**DOI:** 10.3390/s17040716

**Published:** 2017-03-29

**Authors:** Yangzhou Chen, Yuqi Guo, Ying Wang

**Affiliations:** 1 College of Metropolitan Transportation, Beijing University of Technology, Beijing 100124, China; guoyuqi@emails.bjut.edu.cn (Y.G.); wangyingwy@emails.bjut.edu.cn (Y.W.); 2 Beijing Key Laboratory of Transportation Engineering, Beijing 100124, China; 3 Beijing Collaborative Innovation Center for Metropolitan Transportation, Beijing 100124, China

**Keywords:** urban freeway network, dynamic graph hybrid automata, piecewise affine linear system, switched state observer, density estimation

## Abstract

In this paper, in order to describe complex network systems, we firstly propose a general modeling framework by combining a dynamic graph with hybrid automata and thus name it Dynamic Graph Hybrid Automata (DGHA). Then we apply this framework to model traffic flow over an urban freeway network by embedding the Cell Transmission Model (CTM) into the DGHA. With a modeling procedure, we adopt a dual digraph of road network structure to describe the road topology, use linear hybrid automata to describe multi-modes of dynamic densities in road segments and transform the nonlinear expressions of the transmitted traffic flow between two road segments into piecewise linear functions in terms of multi-mode switchings. This modeling procedure is modularized and rule-based, and thus is easily-extensible with the help of a combination algorithm for the dynamics of traffic flow. It can describe the dynamics of traffic flow over an urban freeway network with arbitrary topology structures and sizes. Next we analyze mode types and number in the model of the whole freeway network, and deduce a Piecewise Affine Linear System (PWALS) model. Furthermore, based on the PWALS model, a multi-mode switched state observer is designed to estimate the traffic densities of the freeway network, where a set of observer gain matrices are computed by using the Lyapunov function approach. As an example, we utilize the PWALS model and the corresponding switched state observer to traffic flow over Beijing third ring road. In order to clearly interpret the principle of the proposed method and avoid computational complexity, we adopt a simplified version of Beijing third ring road. Practical application for a large-scale road network will be implemented by decentralized modeling approach and distributed observer designing in the future research.

## 1. Introduction

A traffic flow network is a typical example of a complex network system [1,2]. Dynamic modeling of the traffic flow network is quite a challenging task in traffic flow analysis and control. Various traffic flow models have been proposed to study complex traffic phenomena on a road network, e.g., as the mathematically most consistent macroscopic traffic flow, the well-known LWR model proposed by Lighthill, Whitham [3] and Richards [4], which formulates the relationship among the key traffic flow parameters such as density, flow etc. using continuous time partial differential equations. The second-order model is presented by Payne [5]. From the view of application, Daganzo [6,7] first proposed the Cell Transmission Model (CTM) by spatially and temporally discretizing the LWR model. Later, many researchers further improved the CTM. For example, Lebcque [8] considered the merge law and the diverge law of traffic flow according to the proportional distributions rule. Fl$\ddot{o}$tter$\ddot{o}$d and Nagel [9] developed the model by considering the merge and diverge law of multiple cells. Gomes and Horwitz [10,11,12] proposed the Asymmetric Cell Transmission Model (ACTM) and applied it to the freeway on-ramp metering control. Lo and Lin [13,14] amended the CTM to describe road networks with intersections. Sumalee et al. [15] developed the Stochastic Cell Transmission Model (SCTM). Canudas-de-Wit [16] proposed a graph constrained CTM.

These modified CTMs mainly focus on rules of transmitted traffic flow but give too little care to the description of road network structure, which leads to a lack of modularization and expandability in modeling a large-scale traffic network. Moreover, the nonlinear relationship between traffic flow and density results in study difficulty. Although Mu$\tilde{n}$oz and Sun et al. [17,18] presented the Switching Mode Model (SMM) which is a piecewise linearized version of the CTM, this model may be improved further by introducing some modeling tools as solid theoretical basis. Hence, in the previous work [19,20,21] we proposed a model framework by introducing dynamic digraph and hybrid automata. Different from the traditional digraph method describing the road topology [22], we adopted dual digraph idea and introduced dynamic digraph [23] into the description of traffic flow over a road network, where road segments are marked vertex, and traffic flow directions are considered as the directed edges of digraph. In our opinion, it has at least two advantages. Firstly, dynamic weights of describing traffic state in a road segment can be added in vertices as customary as in the weight digraph theory. Secondly, traffic flow directions can be clearly indicated by the directed edges and transmitted volumes can be expediently described by dynamic weights and switchings on edges, where the edge switchings between vertices can describe the traffic light signals.

On the other hand, we point out that hybrid automata, as a kind of hybrid dynamic system models [24,25], are an appropriate model to describe a multi-mode dynamic process. For instance, references [26,27] applied the hybrid automaton theory to automated highway systems (AHS). Lei and Ozguner [28] presented a hybrid automaton model for a single intersection for the first time. Chen et al. [29,30,31] studied the optimal control of single intersection and the cooperative control between intersections by using hybrid automata. Mu$\tilde{n}$oz and Sun et al. [17,18] presented the Switching Mode Model (SMM) for the California Freeway. In the model framework proposed in [19,20,21] we introduced hybrid automata to describe traffic densities in road segments.

Consequently, by combining dynamic digraph with hybrid automata and applying the parallel composition law between hybrid automata [32,33], we can obtain a new networked model and name it Dynamic Graph Hybrid Automata (DGHA). As an independent model framework, the DGHA can be applied in various dynamic network systems with multi-mode switchings. When applying it to model traffic flow of a road network, one can adopt different types of traffic variables as the state of the hybrid automata, such as two-dimensional variables of traffic density and vehicle average velocity. One may also apply, in the place of the hybrid automata, more complex uncertain models, and more modes taken when piecewise linearizing the nonlinear fundamental diagram. However, in this paper, as well as in [19,20,21], we embedded CTM into DGHA and thus only the traffic density variable was used as the state.

In this paper, we first further improve the DGHA framework proposed in [19,20,21] and the modeling procedure of urban freeway networks. More in detail, we first restate the definition of DGHA model more clearly. Then we analyze types and number of combination modes of cells (the partitioned road segments) when embedding CTM into the DGHA. Compared with the existing literature [34], we not only consider the multi-mode switchings of vertex state, but also take into account the multi-mode switchings of edge weights. In other words, the nonlinear expression of the density in a cell is described by a linear hybrid automaton and the nonlinear expression of the transmitted traffic flow between two cells is transformed into piecewise linear functions in terms of multi-mode switchings. We distinguish two modes (free flow and congested flow) in each cell and two modes (upward traffic wave and downward traffic wave) for each of the three types of connections between cells (linear connection, merge connection and diverge connection). Although the number of the combination modes is larger than that in the existing literature [34], e.g., about 5400 possible combination modes for a road partitioned into 10 cells (see the proof in Section 3.5), the combination modes not only describe the traffic state in a road segment but also the relative flow feature between two road segments. Next we give a convex polyhedral description of the combination modes by introducing a partition of state subspace. Finally we deduce an expression of Piecewise Affine Linear System (PWALS) for the whole freeway network. We point out emphatically that the modeling procedure is modularized and rules-based. Thus we develop an algorithm to implement modeling procedure of traffic networks by accomplishing parallel composition of cells with the help of a computer program. So compared with the traditional manual calculation [17,18,34], the modeling procedure is easily-extensible with the help of the algorithm and can be applied to traffic networks with arbitrary topology structures and sizes, including the road networks with signal intersections.

The proposed PWALS model can be utilized in many aspects, such as traffic state estimation and traffic flow control. In this paper, we focus on the estimation problem of traffic densities. Over the past decades, abundant work on this problem has been done by using different approaches, including the Kalman filter [22,35,36,37], particle filtering [38], the set-valued estimation [39,40,41]. These approaches are not based on networked hybrid automata and thus cannot be directly applied to our model.

On the other hand, the simpler state observer has been well-known as a powerful estimation tool. However, for the DGHA, we need to consider a switching observer. In the existing literature, the design approaches of switching observer can be divided into two classes, depending on whether the active modes are known or not. When the active modes are known, one can utilize the switched Luenberger-type observer with the same switching rules as in the model. The corresponding observer gains can be found by using the Lyapunov function approach for the switched dynamics of estimation error system and solving Linear Matrix Inequalities (LMI) [42,43,44,45,46]. Literature [47] proposed a switching observer for the systems with continuous state jumps and based on a known and fixed switching signal sequence. In the case when the active modes are unknown, meanwhile one is required to identify the modes on the basis of the observation of the output of the system over a certain interval. Pettersson [48,49] proposed multiple quadratic Lyapunov functions to design observers for a switched linear system with continuous state jumps. Literature [50] also deals with the observer design problem for continuous-time switched linear systems with unknown switchings.

The authors in [16] proposed a graph constrained CTM observer. In [51] they further introduced a robust mode selector for the uncertain graph-constrained switching mode model and applied it to highway traffic density estimation via a switched state observer. Alvarez-Icaza et al. [52] designed adaptive observer to estimate vehicle density.

As to the sufficient condition of observer design, the system must be observable, this problem is closely related to the traffic sensor placement in the traffic network. References [53,54] presented an algebraic approach to understand the problem of identifying which subsets of OD-pair and link flows can be calculated based on a given subset of observed OD-pair and link flows, and further applied the algorithm on the Nguyen-Dupuis network problem. A new framework which investigates observability in terms of flow and routing information on network arcs was proposed in [55].

In this paper, we focus on the design of a switched state observer based on the deduced PWALS model to estimate traffic densities under the assumption that the active modes are arbitrary but known. We will discuss more practical issues when the active modes are unknown in the future research.

The main contributions of the paper can be summarized in the following two aspects: (1) The DGHA modeling framework is proposed and a PWALS model is deduced when embedding the well-known CTM into the DGHA. Particularly, this modeling procedure is modularized and easily-extensible and can be used to model traffic networks with arbitrary topology structures and sizes; (2) A switched state observer is designed to estimate traffic densities of an urban freeway network. The corresponding observer gains are found by using the Lyapunov function approach for the switched dynamics of estimation error system and solving Linear Matrix Inequalities (LMI).

The rest of the paper is organized as follows. In Section 2, we provide some relevant background material about dynamic graph and hybrid automata and propose the modeling framework of Dynamic Graph Hybrid Automata. In Section 3, the DGHA framework is used to model traffic flow network by embedding the CTM into it. In Section 4, we deal with the design of the switched state observer. In Section 5, we illustrate the obtained results by applying them to the simplified version of Beijing third ring road. The paper is concluded in Section 6.

## 2. Preliminaries

First of all, we briefly review dynamic graph theory [23] and hybrid automata theory [25] and then combine dynamic digraph with hybrid automata to propose a new model framework which we call Dynamic Graph Hybrid Automata (in short, DGHA) [19,20].

### 2.1. Dynamic Graph

A *graph* [23] is a pair (V,E), where *V* is a finite set of vertices or nodes, and *E* is a set of undirected edges, each being an unordered pair $\left\{i,j\right\}$ and expressing a link between two vertices i,j∈V. A *digraph* is a pair (V,E), where *V* is a finite set of vertices or nodes, and *E* is a set of directed edges $e_{ij}\in V\times V,i\neq j$, expressing a link from vertex i∈V to vertex j∈V.

A *vertex-weighted graph or digraph* is a triple $\left(V,E,F\right)$, where (V,E) is a graph or digraph, F:V→X is a function to assign a weight $x_{i}\in X$ for every vertex *i*, and *X* is a pre-specified set. An *edge-weighted graph or digraph* is a triple $\left(V,E,G\right)$, where (V,E) is a graph or digraph, G:E→Y is a function to assign a weight $y_{ij}$ for every edge $\left\{i,j\right\}$ or $e_{ij}$, and *Y* is a pre-specified set. A *fully-weighted graph or digraph* $\left(V,E,F,G\right)$ has weights assigned to both vertices and edges. Depending on the context, the weight sets X,Y may be real numbers, complex numbers, integers, or even elements of a group or a field, etc.

The set *V* of vertices of a graph or digraph (V,E) is said to be dynamic if its number |V| is time-varying. The set *E* of edges of a graph or digraph (V,E) is said to be dynamic if the edges are time-varying on-off switches. The vertex-weight function (respectively, edge-weight function) is called dynamic if the weights $x_{i}$ (respectively, $y_{ij}$) are time-varying. A *fully-weighted graph* or a *digraph* is called to be *dynamic* when any one of the four entities $\left(V,E,F,G\right)$ is dynamic.

**Remark** **1.** Dynamic set V of vertices means that some vertices may be added or removed. When a vertex is added or removed, the corresponding edge linking is also appended or eliminated. Dynamic set E of edges implies that edges may be added or deleted over time. We also say that the graph or digraph has a switched set of edges.

**Remark** **2.** In what follows, for a digraph (V,E), we denote by $\mathrm{Pre}\left(i\right)=\left\{l\in V:e_{li}\in E\right\}$ the set of all upstream neighbouring vertices and $\mathrm{Post}\left(i\right)=\left\{j\in V:e_{ij}\in E\right\}$ the set of all downstream neighbour ones of vertex i, respectively. Thus $N_{i}=\mathrm{Pre}\left(i\right)\cup \mathrm{Post}\left(i\right)$ is the set of all neighbour vertices of vertex i. We also introduce notation ${\bar{N}}_{i}=\left\{i\right\}\cup N_{i}$.

### 2.2. Hybrid Automaton

A *hybrid automaton [25] with continuous input and output* *H* is a collection $H=\left(S,\mathrm{Init},U,f,\delta ,\mathrm{Inv},G,R,Y,g\right)$, where

- S=Q×X is a state space of hybrid state variables $\left(q,x\right)$, where *Q* is a finite set of discrete states *q* and $X\subseteq R^{n}$ is a *n*-dimensional state space of continuous state vector *x*;
- Init⊆S is a set of initial hybrid states;
- $U\subseteq R^{m}$ is a set of continuous input variable *u*;
- f:S→X is a vector field describing the continuous state dynamics defined by
  $$\begin{array}{c} x\left(t+1\right)=f(q\left(t\right),x\left(t\right),u\left(t\right)); \end{array}$$
- δ:S→Q is a transition map describing the discrete state dynamics. One can also use a binary relation set Δ=Q×δ(S)⊆Q×Q to express the discrete state transition;
- $\mathrm{Inv}:Q\to 2^{X}$ defines the domain of continuous state vector under each discrete state. The domain is also called invariant set of continuous state vector;
- $G:\Delta \to 2^{X}$ defines a guard condition for each discrete state transition;
- R:Δ→X is a reset map to assign a new initial state to the continuous state variable after the transition of the discrete state.
- $Y\subseteq R^{p}$ is a set of continuous output variable *y*;
- g:S→Y is an output map defined as
  $$\begin{array}{c} y\left(t\right)=g(q\left(t\right),x\left(t\right)); \end{array}$$

**Figure 1:**
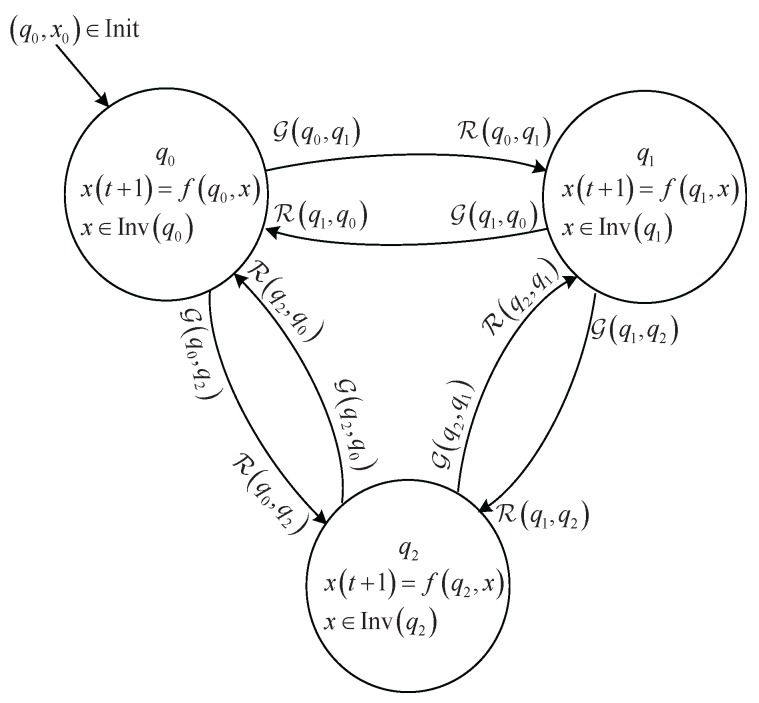
Digraph expression of hybrid automaton.

A hybrid automaton can also be expressed by a digraph as shown in Figure 1 [25]. We notice that in this paper we focus on discrete time system defined by a difference equation for the continuous part of the hybrid automaton.

### 2.3. Dynamic Graph Hybrid Automata

Now we combine a dynamic graph with hybrid automata to propose a new model which we call Dynamic Graph Hybrid Automata (DGHA, for short). An early version of the model was given in references [19,20,21]. In what follows, we restate it in the way of parallel composition of hybrid automata.

**Definition** **1.** *DGHA model is a hybrid automaton H over a digraph (V,E) which described by the following four parts:*

(1) Fully weighted dynamic digraph. *In $G=\left(V,E,H,\Phi \right)$, $V=\left\{1,\cdots ,N\right\}$ is a finite set of N fixed vertices, and $E=\left\{e_{ij}:i,j\in V\right\}$ is a set of directed edges, H and* Φ *are sets of the automata and the functions that assign the vertex weights and edge weights, respectively, which are described in detail as follows;*
(2) Vertex dynamics. *The elements in $H=\left\{H_{i}:i\in V\right\}$ are hybrid automata $H_{i}=\left(S_{i},\mathrm{Ini}t_{i},U_{i},f_{i},\delta_{i},{\mathrm{Inv}}_{i},G_{i},R_{i},Y_{i},g_{i}\right)$ to describe the dynamics of vertex i∈V, whose components are similarly defined as in Subsection B.*
(3) Edge weight functions. *The set $\Phi =\left\{\phi_{ij}:e_{ij}\in E\right\}$ consists of the functions $\phi_{ij}:Y_{i}\times Y_{j}\to R^{m}$ that assign a weight $\phi_{ij}\left(y_{i},y_{j}\right)$ for each edge $e_{ij}\in E$;*
(4) Composition of hybrid automata. *In $\mathrm{DGHA}=\left(V,E,H\right)$, $H=\left(S,\mathrm{Init},f,\delta ,\mathrm{Inv},G,R\right)$ is the closed hybrid automaton over the digraph (V,E) obtained by parallel composition of hybrid automata in H, i.e., S=Q×X with $Q=Q_{1}\times \cdots \times Q_{N}$ and $X=X_{1}\times \cdots \times X_{N}$; $\mathrm{Init}=\mathrm{Ini}t_{1}\times \cdots \times {\mathrm{Init}}_{N}$; f:S→X is defined as $f={\bar{f}}_{1}\times \cdots \times {\bar{f}}_{N}$ and ${\bar{f}}_{i}:S{|}_{i}\to X_{i}$ describes the dynamics of vertex i by substituting $u_{i}\left(t\right)$ in the the following equation*
  (1) $$\begin{array}{c} x_{i}\left(t+1\right)=f_{i}(q_{i}\left(t\right),x_{i}\left(t\right),u_{i}\left(t\right)), \end{array}$$
  *with the following relation:*
  (2) $$\begin{array}{c} u_{i}\left(t\right)=\sum_{l\in \mathrm{Pre}(i)}\phi_{li}(y_{l}\left(t\right),y_{i}\left(t\right))-\sum_{j\in \mathrm{Post}(i)}\phi_{ij}(y_{i}\left(t\right),y_{j}\left(t\right)), \end{array}$$
  *where ${S|}_{i}$ denotes the projected subspace of S on the state components with indices in ${\bar{N}}_{i}$, and for any i∈V the output $y_{i}$ is given by*
  (3) $$\begin{array}{c} y_{i}\left(t\right)=g_{i}(q_{i}\left(t\right),x_{i}\left(t\right)); \end{array}$$
  *transition map δ:S→Q is defined by the following rule:*
  $$\delta \left(s_{1},\cdots ,s_{N}\right)=\left({q^{\prime }}_{1},\cdots ,{q^{\prime }}_{N}\right)\Leftrightarrow \delta_{i}\left(s_{i}\right)={q^{\prime }}_{i},\forall i\in V;$$
  *$\mathrm{Inv}=\mathrm{In}v_{1}\times \cdots \times \mathrm{In}v_{N}$; $G=G_{1}\times \cdots \times G_{N}$; $R=R_{1}\times \cdots \times R_{N}$.*

**Figure 2:**
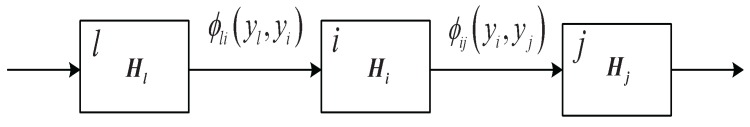
A general DGHA model.

A DGHA model can also be expressed by a digraph as shown in Figure 2.

**Remark** **3.** In Definition 1, the vertex set V and edge set E are assumed to be fixed. However, instead of the fixed edge set E, one can use a dynamically switching edge set $E\left(t\right)=\left\{\delta_{ij}\left(t\right)e_{ij}:i,j\in V\right\}$ by introducing a Boolean function $\delta_{ij}:R\to \left\{0,1\right\}$ to indicate appearing or disappearing of edges, which can be used to express the traffic lights, i.e., $\delta_{ij}\left(t\right)=0$ if the light is red, and $\delta_{ij}\left(t\right)=1$ if the light is green. In this case, the edge weight function will be also affected by the switching function and thus become $\delta_{ij}\left(t\right)\phi_{ij}\left(y_{i}\left(t\right),y_{j}\left(t\right)\right)$. In this paper, we focus on modeling urban freeway, so we do not further discuss this kind of models more in detail.

**Remark** **4.** In general, the functions $\phi_{ij}\left(y_{i},y_{j}\right)$ are nonlinear. When the hybrid automata of vertices are all linear or affine linear, one can further express these functions by linear ones in a way of multiple mode switchings. In this case, the vertex hybrid automata may be changed accordingly, depending on the edge modes and the modes of all neighbour vertices (see the application below in traffic flow model).

## 3. DGHA Model For Urban Freeway Network

As a generalized model framework, the DGHA can be applied to various complex network systems in different fields. When applying it to a traffic flow network, one can adopt different types of traffic variables to describe traffic state in road segments, e.g., two-dimensional traffic variables of traffic densities and vehicle average velocity. But in this paper we apply the DGHA to model traffic flow of an urban freeway network by embedding the well-known CTM into the DGHA. That means we only use the traffic densities to describe traffic state in road segments. As for urban street networks with intersections, we need DGHA with switching edges to describe traffic lights, which goes beyond our research in this paper.

We improve our previous work in [19,20,21] in the following aspects. Firstly, we introduce a partition of state subspace such that the combination modes of cells can be described by convex polytopes. Meanwhile, by adopting parallel composition algorithm of automata to obtain a hybrid automaton with high dimension over the whole road network. We finally deduce a Piecewise Affine Linear System (PWALS) of the overall road network. Based on the obtained PWALS, we further analyze the types and number of combination modes of cells and give a corresponding computational formula of the number of modes. An algorithm is developed on the basis of DGHA framework, and one can apply this algorithm to implement modelling of traffic networks with arbitrary topology structures and sizes.

### 3.1. The Dual Digraph Description of Road Network

In most literature, a road network is modeled as a digraph according to its original structure [22], i.e., each intersection or bottleneck point is represented as a vertex and the road section between adjacent intersections or bottleneck points is represented as the directed edge. However, in contrast to this traditional methodology, in our DGHA model, we adopt the dual digraph of the original structure of the road network. More precisely, each road section connecting two adjacent intersections or bottleneck points is partitioned into one or several segments and these segments, which are called cells, are modeled as vertices of the digraph. Two vertices are connected by an directed edge if there is traffic flow transition between them along the arrowed direction.

In our opinion, the dual digraph description has at least three advantages. Firstly, the network is easily extended by adding vertices and edges of the digraph. Secondly, weights describing dynamic procedures can be added in vertices as customary as in the weight digraph theory. Finally, traffic flow directions can be clearly indicated by the directed edges of the digraph and transited flows can be expediently described by dynamic weights and switchings, and especially, the edge switchings between vertices can describe the traffic light signals.

In this paper, we use fully weighted dynamic digraph $G=\left(V,E,H,\Phi \right)$ to describe urban freeway network, where $V=\left\{1,2,\cdots ,N\right\}$ denotes the set of all partitioned road segments of a given road network, $E=\left\{e_{ij}:i,j\in V\right\}$ denotes the set of all the directed edges indicating the transition of traffic flow, and the set H of automata and the set Φ of edge weighted functions will be illustrated later. Compared with the traditional modeling method, directed edges clearly indicate the transition of traffic flows and their weights express the traffic volumes transited during a pre-specified time interval. For the description of urban street network with intersections, we can express the switchings of traffic lights, instead of the fixed set *E*, by adopting a switching edge set E(t).

### 3.2. Dynamics of Traffic Flow in Road Segment

**Figure 3:**
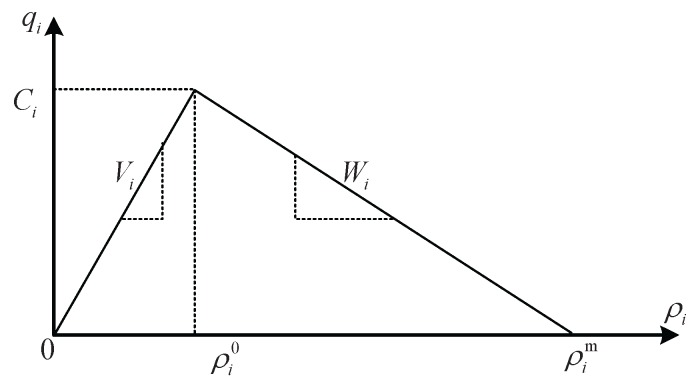
Triangular fundamental diagram.

To the best of our knowledge, the CTM [6,7] can well describe traffic flow, where the triangular fundamental diagram or the trapezoid fundamental diagram is adopted to approximatively express flow-density relationship [17,18,22]. In this paper, we use traffic density $\rho_{i}$ to express the traffic state of road segment *i*, and also adopt the triangular fundamental diagram shown in Figure 3 for road segment *i*, where $C_{i}$ is the traffic capacity (vph), $V_{i}$ is the free flow speed (mph), $W_{i}$ is the traffic wave speed (mph), $\rho_{i}^{0}$ is the critical density (vpm), and $\rho_{i}^{m}$ is the maximum/jam density (vpm).

To express the dynamics of vertex *i*, we use the hybrid automaton $H_{i}=\left(S_{i},{\mathrm{Init}}_{i},U_{i},f_{i},\delta_{i},\mathrm{In}v_{i},G_{i},Y_{i},g_{i}\right)$, whose entries are illustrated as follows:

- $S_{i}=Q_{i}\times X_{i}$, where $Q_{i}=\left\{F,C\right\}$, “F=Freeflow, C=Congestedflow”, and the continuous state space is $X_{i}=R$ since only density $\rho_{i}$ is adopted as continuous state variable;
- ${\mathrm{Init}}_{i}\subset S_{i}$ can be given arbitrarily;
- The input variable $u_{i}$ represents the changed flow of road segment *i* during the time period $\left[tT,(t+1)T\right]$, where *T* is the sample time period. Thus we have the input space $U_{i}=R$;
- The vector field $f_{i}:S_{i}\times U_{i}\to X_{i}$ is described by the following equation:
  (4) $$\begin{array}{c} {\rho_{i}}^{+}=\rho_{i}+a_{i}u_{i}. \end{array}$$
  where $a_{i}=T/L_{i}$ and $L_{i}$ is the length of the road segment (cell) *i*. Here and henceforth for simplicity we omit the time *t* in various variables and use $\rho_{i}^{+}$ to express $\rho_{i}\left(t+1\right)$;
- The transition map $\delta_{i}$ is defined according to the guard conditions, i.e., a transition happens if and only if the corresponding invariant set is damaged and the guard condition is satisfied; The reset map $R_{i}$ is identical and thus is omitted;
- The invariant sets are ${\mathrm{Inv}}_{i}\left(F\right)=\left\{\rho_{i}:0\leq \rho_{i}\leq \rho_{i}^{0}\right\}$ and ${\mathrm{Inv}}_{i}\left(C\right)=\left\{\rho_{i}:\rho_{i}^{0}<\rho_{i}\leq \rho_{i}^{m}\right\}$;
- The guard conditions are $G_{i}\left(F,C\right)=\left\{\rho_{i}:\rho_{i}>\rho_{i}^{0}\right\}$ and $G_{i}\left(C,F\right)=\left\{\rho_{i}:\rho_{i}\leq \rho_{i}^{0}\right\}$;
- The output space is $Y_{i}=R^{2}$;
- The output function $g_{i}:S_{i}\to Y_{i}$ is defined as $y_{i}=g_{i}\left(q_{i},x_{i}\right)={\left[r_{i},\;s_{i}\right]}^{T}$, where $r_{i}\;=\;\min\left\{C_{i},W_{i}\left(\rho_{i}^{m}-\rho_{i}\right)\right\}$ is the flow that can be received by cell *i* over the interval $\left[tT,\left(t+1\right)T\right]$), $s_{i}=\min\left\{V_{i}\rho_{i},C_{i}\right\}$ is the flow that can be supplied by cell *i* over the interval $\left[tT,\left(t+1\right)T\right]$), i.e.,
  (5) $$\begin{array}{cc} & y_{i}=g_{i}\left(F,\rho_{i}\right)=\left[\begin{array}{c} C_{i}\\ V_{i}\rho_{i} \end{array}\right], \end{array}$$
  (6) $$\begin{array}{c} y_{i}=g_{i}\left(C,\rho_{i}\right)=\left[\begin{array}{c} W_{i}\left(\rho_{i}^{m}-\rho_{i}\right)\\ C_{i} \end{array}\right]. \end{array}$$

### 3.3. Multi-Mode Description of Transited Traffic Flows

The edge weight function $\phi_{ij}:Y_{i}\times Y_{j}\to R$ in the set $\Phi =\left\{\phi_{ij}:e_{ij}\in E\right\}$ expresses traffic flow practically transited from upstream cell *i* to downstream cell *j*. According to the rule of the CTM, the transited traffic flow is defined by a nonlinear function

(7) $$\begin{array}{c} \phi_{ij}\left(y_{i},y_{j}\right)=\min\left\{s_{i},r_{j}\right\}, \end{array}$$

where $r_{j}$ is the flow that can be received by cell *j* and $s_{i}$ is the flow that can be supplied by cell *i*, both during the time interval $\left[tT,\left(t+1\right)T\right]$.

In what follows, we give a two-mode switching description of linear functions via two modes “D = Downward wave, U = Upward wave”:

(8) $$\begin{array}{c} D:\;\phi_{ij}\left(y_{i},y_{j}\right)=s_{i},\;\mathrm{if}\;s_{i}\leq r_{j},\\ U:\;\phi_{ij}\left(y_{i},y_{j}\right)=r_{j},\;\mathrm{if}\;s_{i}>r_{j}. \end{array}$$

**Remark** **5.** Two modes “D” and “U” describe the relation of the traffic flow between the road segments i and j. “D” mode means that the road segment j can release the traffic flow from i, while “U” mode indicates that the road segment j blocks the traffic flow from i.

### 3.4. Composition for DGHA Model of Traffic Flow Network

In literature [56], used a combination of LWR model with Godunov based numerical solutions [53] to solve the Riemann problem where the initial condition is a piecewise constant function with two values $\rho_{\ell }$ and $\rho_{r}$ for the upstream (left) and downstream (right) densities. Either a shockwave or a rarefaction wave originates from the junction of the two densities. A shockwave develops if $f^{\prime }\left(\rho_{\ell }\right)>f^{\prime }\left(\rho_{r}\right)$. Similarly, a rarefaction develops if $f^{\prime }\left(\rho_{\ell }\right)<f^{\prime }\left(\rho_{r}\right)$. However, in the paper the hybrid switching automata approach proposed is to solve the traffic characteristics and system evolution by considering a Riemann problem with a piecewise constant initial condition.

Now we discuss the composition rules of the vertex automata affected by the edge weight functions described in Section 3.2 and Section 3.3 above. That is, we combine the dynamics of traffic flows in road segments with the transited traffic flows described by the two-mode switching linear functions to deduce the DGHA model of traffic flow network.

We assume that the merging interrelationship of flows in segments l∈Pre(i) and dividing interrelationship of flows in segments j∈Post(i) have been separated according to the rules via merging or dividing ratio [6,8], so that we can independently deal with the transited flows between any two segments (l,i) or (i,j). In other words, assuming that $\zeta_{li}$ and $\eta_{ij}$ are the merging and dividing ratios respectively, the Equation (7) is modified by the merging and dividing rules

(9) $$\begin{array}{c} \phi_{li}\left(y_{l},y_{i}\right)=\min\left\{s_{l},\zeta_{li}r_{i}\right\}, \end{array}$$

(10) $$\begin{array}{c} \phi_{ij}\left(y_{i},y_{j}\right)=\min\left\{\eta_{ij}s_{i},r_{j}\right\}. \end{array}$$

Thus, according to the composition rules of hybrid automata in Equation (2), the function $u_{i}$ in Equation (4) is given as follows:

(11) $$\begin{array}{c} u_{i}=\sum_{l\in \mathrm{Pre}(i)}\min\left\{s_{l},\zeta_{li}r_{i}\right\}-\sum_{j\in \mathrm{Post}(i)}\min\left\{\eta_{ij}s_{i},r_{j}\right\}. \end{array}$$

Especially, when only linear connections exist, e.g., in a urban ring freeway, we have $\mathrm{Pre}\left(i\right)=\left\{l\right\}$ and $\mathrm{Post}\left(i\right)=\left\{j\right\}$ in Equation (11), and thus $\zeta_{li}=1$ and $\eta_{ij}=1$, which is the case we assume for simplicity in the following statement.

Now we use the way of modes in Equation (8) to express the Equation (11) into a switching procedure between a series of linear equations. In other words, we analyze the modes of vertex *i* in connected network by combining discrete states $Q_{i}$ and modes of all the edges connected with vertex *i*. It is obvious that these modes depend on the states of the vertices in ${\bar{N}}_{i}$, and each mode can be expressed by a group of inequalities of state variables $\rho_{k},k\in {\bar{N}}_{i}$. Thus, we use a partition of state subspace $\Theta_{i}=\prod_{k\in {\bar{N}}_{i}}X_{k}$ to express these modes so that the Equation (4) with Equation (11) is expressed by a piecewise affine linear system (PWALS).

For the special case of $\mathrm{Pre}\left(i\right)=\left\{l\right\}$ and $\mathrm{Post}\left(i\right)=\left\{j\right\}$, Equation (11) into Equation (4) gives the following equation:

(12) $$\begin{array}{c} \rho_{i}^{+}=\rho_{i}+a_{i}(\min\left\{s_{l},r_{i}\right\}-\min\left\{s_{i},r_{j}\right\}) \end{array}$$

In order to Equation (12) into a PWALS, we partition the subspace $\Theta_{i}=\left\{{\bar{\rho }}_{i}={\left(\rho_{l},\rho_{i},\rho_{j}\right)}^{T}\in R^{3}\right\}$ into a group of subsets defined by convex polytopes $\Theta_{ik}=\left\{{\bar{\rho }}_{i}:H_{ik}{\bar{\rho }}_{i}\leq h_{ik}\right\}$, and thus in each of the subsets the flows $q_{li}=\min\left\{s_{l},r_{i}\right\}$ and $q_{ij}=\min\left\{s_{i},r_{j}\right\}$ can be expressed by linear relations.

First of all, we show that the cell *i* generally possesses seven modes with relation to upstream *l* shown in Table 1, where $\alpha_{li}=\min\left\{C_{i}/V_{l},\rho_{l}^{0}\right\}$ and $\beta_{li}=\max\left\{\rho_{i}^{0},\rho_{i}^{m}-C_{l}/W_{i}\right\}$.

As seen in Table 1, the practical modes depend on the traffic capacities $C_{l},C_{i}$. In fact, we can list the following three cases: $C_{l}>C_{i}$, $C_{l}<C_{i}$ and $C_{l}=C_{i}$. In the case of $C_{l}>C_{i}$, there exist six combined modes “FDF, FUF, CUF, CUC, FUC, FDC”; In the case of $C_{l}<C_{i}$, there exist six combined modes “FDF, CDF, CDC, CUC, FUC, FDC”; In the case of $C_{l}=C_{i}$, mode “FUF” (resp. “CDC”) will be merged into mode “FDF” (resp. “CUC”) and thus there exist only five combined modes “FDF, CDF, CUC, FUC, FDC”, where “FDF” and “FDC” indicate free flow, “CUC” and “FUC” are congested flow, and “CDF” is saturated flow. Similarly, one can analyze the modes of *i* with respect to downstream *j*.

Next, we combine the modes of *i* with relation to *l* and *j* to obtain the modes of cell *i* in the connected network by using the corresponding subsets $\Theta_{ik}$. In this case, Equation (12) is expressed into a piecewise affine linear system with the following twelve linear subsystems:

(13) $$\begin{array}{ccc} \rho_{i}^{+} & = & \left(1-a_{i}V_{i}\right)\rho_{i}+\left(a_{i}V_{l}\right)\rho_{l},\\ & & \mathrm{if}\;“\mathrm{FDFDF}”:\;H_{i1}{\bar{\rho }}_{i}\leq h_{i1};\\ & & \mathrm{or}\;\mathrm{if}\;“\mathrm{FDFDC}”:\;H_{i2}{\bar{\rho }}_{i}\leq h_{i2};\\ \rho_{i}^{+} & = & \rho_{i}+\left(a_{i}V_{l}\right)\rho_{l}+\left(a_{i}W_{j}\right)\rho_{j}-a_{i}W_{j}\rho_{j}^{m},\\ & & \mathrm{if}\;“\mathrm{FDFUC}”:\;H_{i3}{\bar{\rho }}_{i}\leq h_{i3};\\ & & \mathrm{or}\;\mathrm{if}\;“\mathrm{FDCUC}”:\;H_{i4}{\bar{\rho }}_{i}\leq h_{i4};\\ \rho_{i}^{+} & = & \rho_{i}+\left(a_{i}V_{l}\right)\rho_{l}-a_{i}C,\\ & & \mathrm{if}\;“\mathrm{FDCDF}”:\;H_{i5}{\bar{\rho }}_{i}\leq h_{i5};\\ \rho_{i}^{+} & = & \left(1-a_{i}V_{i}\right)\rho_{i}+a_{i}C,\\ & & \mathrm{if}\;“\mathrm{CDFDF}”:\;H_{i6}{\bar{\rho }}_{i}\leq h_{i6};\\ & & \mathrm{or}\;\mathrm{if}\;“\mathrm{CDFDC}”:\;H_{i7}{\bar{\rho }}_{i}\leq h_{i7};\\ \rho_{i}^{+} & = & \rho_{i}+\left(a_{i}W_{j}\right)\rho_{j}+a_{i}\left(C-W_{j}\rho_{j}^{m}\right),\\ & & \mathrm{if}\;“\mathrm{CDFUC}”:\;H_{i8}{\bar{\rho }}_{i}\leq h_{i8};\\ \rho_{i}^{+} & = & \left(1-a_{i}W_{i}\right)\rho_{i}+a_{i}\left(W_{i}\rho_{i}^{m}-C\right),\\ & & \mathrm{if}\;“\mathrm{CUCDF}”:\;H_{i9}{\bar{\rho }}_{i}\leq h_{i9};\\ & & \mathrm{or}\;\mathrm{if}\;“\mathrm{FUCDF}”:\;H_{i(10)}{\bar{\rho }}_{i}\leq h_{i(10)};\\ \rho_{i}^{+} & = & \left(1-a_{i}W_{i}\right)\rho_{i}+\left(a_{i}W_{j}\right)\rho_{j}+a_{i}\left(W_{i}\rho_{i}^{m}-W_{j}\rho_{j}^{m}\right),\\ & & \mathrm{if}\;“\mathrm{FUCUC}”:\;H_{i(11)}{\bar{\rho }}_{i}\leq h_{i(11)};\\ & & \mathrm{or}\;\mathrm{if}\;“\mathrm{CUCUC}”:\;H_{i(12)}{\bar{\rho }}_{i}\leq h_{i(12)}. \end{array}$$

where the matrices $H_{ik},h_{ik},k=1,\cdots ,12$ are listed in Appendix A to express $\Theta_{ik}=\left\{{\bar{\rho }}_{i}:H_{ik}{\bar{\rho }}_{i}\leq h_{ik}\right\}$. We add $\Theta_{i0}=\Theta_{i}\backslash \cup_{k=1}^{12}\Theta_{ik}$, and thus have $\Theta_{i}=\cup_{k=0}^{12}\Theta_{ik}$.

Similarly, we can deal with all the cells in an urban ring freeway which is partitioned into *N* cells. Finally, we can obtain all modes of the freeway network by finding intersections of all the convex polytopes $\Theta_{ik},\;i=1,\cdots ,N,\;k=1,\cdots ,12$, in the state space $R^{N}$.

Let S=S(N) be the number of all the combined modes of the freeway network with *N* cells, and $D_{s}\subset R^{N},s=1,\cdots ,S$, be the convex polytopes describing the combined modes. We use $x\;=\;{\left[\rho_{1},\cdots ,\rho_{N}\right]}^{T}\in R^{N}$ to denote the traffic density vector of all the cells, use the vector $u\in R^{M}$ to represent the traffic demand from on-ramps of the freeway network, and assume that the dividing ratio of the cell *i* with an off-ramp to the downstream cell *j* is $\eta_{ij}$. Thus traffic flow of the freeway network can be modeled by a PWALS with *S* subsystems and a switching function σ:[0,+∞)→{1,2,⋯,S} among these subsystems:

(14) $$\begin{array}{c} x\left(t+1\right)=A_{\sigma (t)}x\left(t\right)+B_{\sigma (t)}u\left(t\right)+F_{\sigma (t)}, \end{array}$$

where the switching function σ(t) is determined by the convex polytopes $D_{s}$, i.e., σ(t)=s if and only if $x\left(t\right)\in D_{s}$; for s=1,⋯,S, vector $F_{s}$, matrices $A_{s}$ and $B_{s}$ consist of the parameters in the fundamental diagrams and the dividing ratios of all the cells.

### 3.5. Analysis of the Number of Combined Modes

As the basis of other work, such as density estimation and on-ramp metering, it is essential to analyze the complexity of combined modes. Now we take an urban freeway with *N* partitioned cells as an example to investigate the combined mode number S(N).

We prove that S(N) satisfies the following relation:

(15) $$\begin{array}{c} S(N)=2S(N-1)+S(N-2),\;N\geq 3, \end{array}$$

with initial conditions S(1)=2 and S(2)=5.

**Figure 4:**
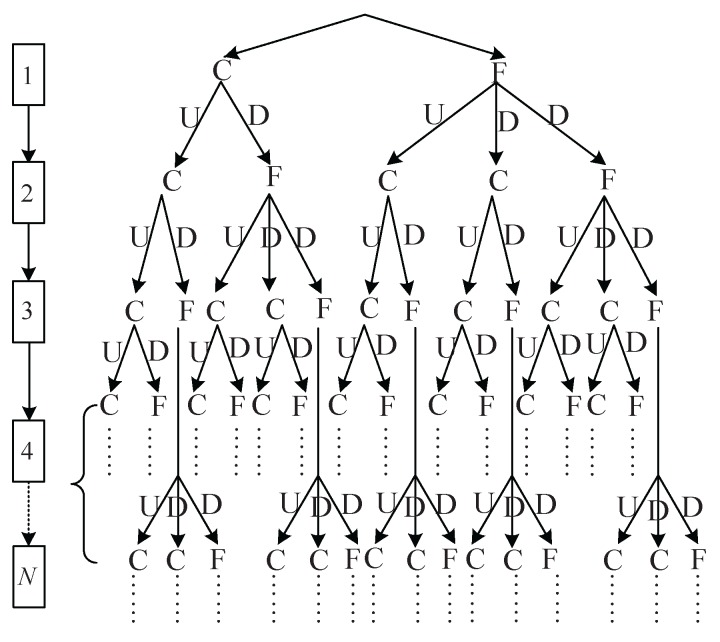
Combination modes.

The proof is as follows. As seen in Figure 4, if the current cell is in mode “F”, there are only three possible modes combined with the downstream cells, i.e., “FDF”, “FUC”, “FDC”. Otherwise, there exist only two possible combination modes when the current cell is in mode “C”, i.e., “CDF”, “CUC”. Hence, we have the following equation:

(16) $$\begin{array}{c} S\left(N\right)=3S_{F}\left(N-1\right)+2S_{C}\left(N-1\right) \end{array}$$

where $S_{F}\left(N-1\right)$ and $S_{C}\left(N-1\right)$ are the mode numbers of the first N−1 cells when the (N−1)th cell is in mode “F” and “C”, respectively.

On the other hand, we have $S_{F}\left(N-1\right)=S\left(N-2\right)$ since “F” can appear after any modes. Moreover, because of $S_{F}\left(N-1\right)+S_{C}\left(N-1\right)=S\left(N-1\right)$, we get $S_{C}\left(N-1\right)\;=\;S\left(N-1\right)\;-\;S_{F}\left(N\;-\;1\right)$. Substituting these two expressions into Equation (16), we obtain the following equation:

$$\begin{array}{ccc} S(N) & = & 3S_{F}\left(N-1\right)+2S_{C}\left(N-1\right)\\ & = & 3S\left(N-2\right)+2\left[S(N-1)-S(N-2)\right]\\ & = & 2S(N-1)+S(N-2), \end{array}$$

and thus verify the Equation (15).

Although we focus on the analysis of urban freeway networks, we can use similar method to investigate other types of road networks. However, since these combination modes are affected by various factors, such as traffic lights, the types and the number of merge connections and diverge connections, the situation may become more complex. On the other hand, we should point out that although we list all the combination modes for a given road network theoretically, further studies are needed to check whether all the modes exist practically and how these modes change during traffic flow evolution.

In the literature [34], the number of modes is $2^{N}$ for a road segment with *N* cells. They only considered the modes in the vertices and ignored the multi-mode switchings of the edges. In our DGHA model, the modes of vertices and edges are both considered. Consequently, the modes are more than the those in [34].

## 4. State Observer Design

State observers have been considered as effective tools of state estimation in the control theory, and various types of state observers have been developed and widely applied in practical fields. In this section, we choose the Luenberger-type switching observer in [43] to estimate traffic densities based on the PWALS Equation (14) for a freeway network divided into *N* cells.

We suppose that *M* fixed or floating sensors are installed on some road segments and aim to estimate the densities on all the segments of the whole freeway network via the PWALS Equation (14) and the traffic data obtained from the sensors.

Let $y\left(t\right)\in R^{M}$ be the measured output vector of the sensors and assume that it has the following relation to the densities:

(17) $$\begin{array}{c} y(t)=Cx(t) \end{array}$$

where *C* is the output matrix depending on the type, number and location of the sensors. We also assume that $\left(A_{s},C\right),s=1,2,\cdots S$, are observable or detectable.

**Figure 5:**
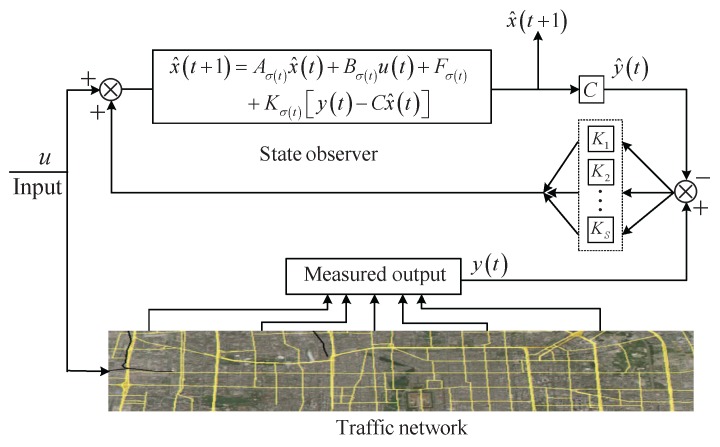
The schematic diagram of state observer.

In this paper, we assume the switching rule of the observer is the same with that of the PWALS Equation (14). Thus, based on the Equation (14) and the measured output Equation (23), we construct the following piecewise affine linear observer (Figure 5):

(18) $$\begin{array}{ccc} \hat{x}\left(t+1\right) & = & A_{\sigma (t)}\hat{x}\left(t\right)+B_{\sigma (t)}u\left(t\right)+F_{\sigma (t)}+K_{\sigma (t)}\left(y\left(t\right)-C\hat{x}\left(t\right)\right), \end{array}$$

where $\hat{x}\left(t\right)\in R^{N}$ is the estimation of the state x(t), $K_{s}$, s=1,⋯,S, are the observer gain matrices which will be designed below, the switching function σ : [0,+∞)→{1,2,⋯,S} is the same with that of the PWALS Equation (14) and thus is determined by the convex polytopes $D_{s},s=1,\cdots ,S$, i.e., σ(t)=s if and only if $x\left(t\right)\in D_{s}$.

Therefore, the state estimation error $e\left(t\right)=x\left(t\right)-\hat{x}\left(t\right)$ can be described by the following switched linear system:

(19) $$\begin{array}{c} e\left(t+1\right)=\left(A_{\sigma (t)}-K_{\sigma (t)}C\right)e\left(t\right). \end{array}$$

In order to ensure $\lim_{t\to \infty }e\left(t\right)=0$ for any switching signal σ(t), the switched linear Equation (19) must be asymptotically stable for any switching signal σ(t). We apply the common quadratic Lyapunov function approach for the following subsystems to compute the observer gain matrices $K_{s}$:

(20) $$\begin{array}{c} e\left(t+1\right)=\left(A_{s}-K_{s}C\right)e\left(t\right),\;s=1,\cdots ,S. \end{array}$$

First of all, we state two well-known lemmas as follows.

**Lemma** **1.** *Assume that $\left(A_{s},C\right),s=1,2,\cdots S$, are observable or detectable. If there exists a positive definite symmetric matrix P satisfying the following Lyapunov inequalities:*

(21) $$\begin{array}{c} \;{\left(A_{s}-K_{s}C\right)}^{T}P\left(A_{s}-K_{s}C\right)-P_{s}<0,\;s=1,\cdots ,S \end{array}$$

*the gain matrices $K_{s},s=1,\cdots ,S$, ensure that, for any switching signal σ(t), the estimation error of the observer Equation (18) exponentially converge to zero.*

**Lemma** **2.** *Let $X_{s}=PK_{s}$. Then the inequalities Equation (21) are equivalent to the following LMIs with respect to P and $X_{s}$:*

(22) $$\begin{array}{c} \left[\begin{array}{cc} P & {\left(PA_{s}-X_{s}C\right)}^{T}\\ PA_{s}-X_{s}C & P \end{array}\right]>0,\;s=1,\cdots ,S. \end{array}$$

Thus, we can find the gain matrices $K_{s}=P^{-1}X_{s},s=1,\cdots ,S$ by solving the LMIs Equation (22).

In fact, the condition in Equation (21) or (22) provides a sufficient and necessary one for the existence of the observer Equation (18). In other words, if the condition Equation (21) or (22) is not satisfied, one has to change the structure of the observer or even to find another type of state estimation approach. The same case happens in other estimation methods such as Kalman filter, where one cannot always find the Kalman gain matrix.

## 5. Application to Beijing Third Ring Freeway

### 5.1. Test Data and Parameter Settings

**Figure 6:**
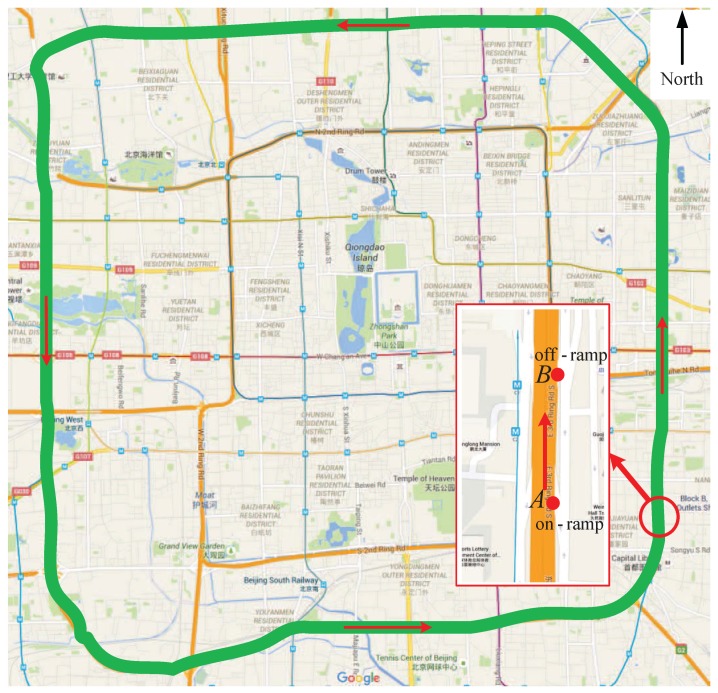
Beijing third ring freeway (from Google Map). The points *A* and *B* are marked as the first on-ramp and the first off-ramp, respectively. The segment between *A* and *B* is labeled as cell 1. (Note: The Chinese word in this map is just the name of some buildings and will not affect the meaning of this image.)

Beijing third ring freeway (Figure 6) is approximately 48 km long and includes 62 on-ramps and 62 off-ramps. We apply the proposed model and state observer to the outer ring freeway. According to consistency rule of cells [21], the outer ring freeway can be partitioned into 128 cells. As shown in Section 3, if a centralized Equation (15) is used for the freeway with 128 cells, there exist $8.44342\times 10^{48}$ combination modes, which results in high computational complexity. Therefore, in this paper, in order to clearly interpret the principle of the proposed method and avoid computational complexity, we take into account a simplified version of the outer ring freeway. For the practical application we will consider a decentralized model and a distributed observer in the future research.

**Figure 7:**
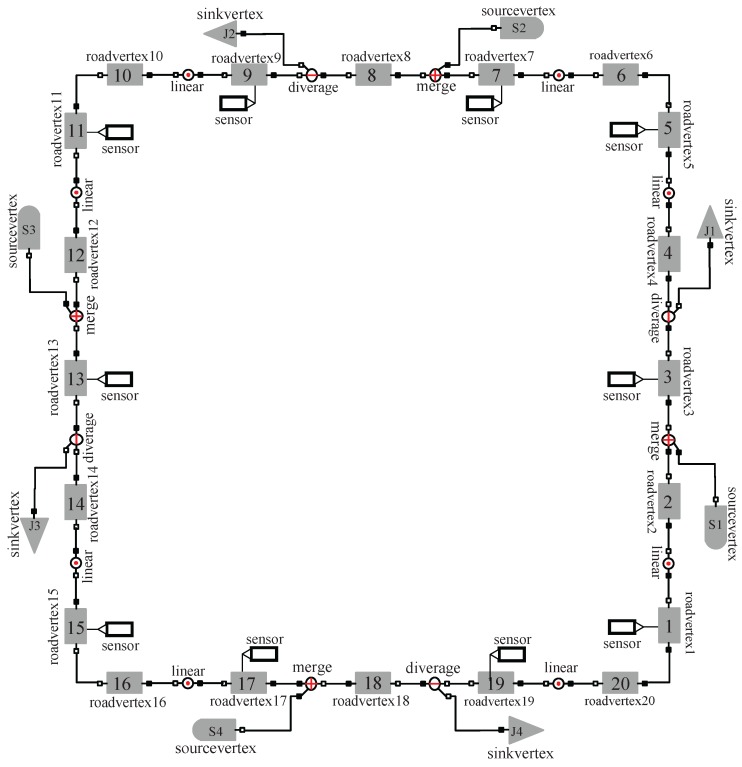
The simplified version of Beijing third ring freeway.

The simplified outer ring freeway (Figure 7) contains 20 cells, 4 on-ramps and 4 off-ramps. Each cell has three lanes and the length of the cells is listed in Table 2. The parameters of all cells are given in Table 3.

Moreover, since Modelica language [20,57] has the advantage of modeling hybrid systems, we choose the open source software OpenModelica to develop a program library to implement the traffic flow model and state estimation.

For the experimental data, we collected 6 h (from 6:00 a.m. to 12:00 a.m.) traffic flow data of 4 on-ramps, 4 off-ramps and traffic densities of 4 road segments of the outer ring freeway on 19 May 2015. Then we used a microscopic model of the outer ring freeway built by Paramics [58] to simulate the traffic flow from 6:00 a.m. to 12:00 a.m. and collect the densities (we call them the simulated densities) by virtual sensors in all the segments, where the real traffic flows of 4 on-ramps and 4 off-ramps were used as the traffic demand and to determine the dividing ratios. The real initial densities of the 4 road segments were used as the initial traffic state of all the cells. In simulation procedure, by setting the dividing ratio, and adjusting the whole microscopic simulator, the modes of traffic flow change in the following sequence order: 20F→16F4C→12F2C2F4C→6F3C3F2C2F4C→6F3C3F8C→16C→7F1C1F3C1F3C3F1C, where kF or kC denotes continuous *k* cells in mode F or C.

The corresponding matrices $A_{s}=\left[a_{i,j}\right]$ and vectors $F_{s}=\left[f_{i}\right]$, s=1,2⋯20, are listed in Appendix B. For the outer ring freeway with 4 on-ramps the matrix $B_{s}=\left[b_{i,j}\right]$ is a constant matrix with the entries $b_{3,1}=0.0149$, $b_{8,2}=0.0116$, $b_{13,3}=0.0109$, $b_{18,4}=0.0238$, and $b_{i,j}=0$ for the other entries in $B_{s}$. Here the sample time period T=5s such that the condition $V_{i}T\leq L_{i},i=1,2\cdots 20$, is satisfied.

Nowadays various sensor technologies can be applied to collect traffic data, including floating vehicle, loop detector, microwave and video. Different source data can provide different measurement outputs and thus one gets different types of matrix *C* in the measurement output Equation (23). One can even utilize the fused data or multi-dimensional data of several types of sensors at the same time.

However, in this paper we used the loop detector to collect traffic densities. In the example, the virtual loop detectors in Paramics installed in the cells 1, 3, 5, 7, 9, 11, 13, 15, 17, 19 were used to collect traffic densities (see Figure 7). Hence, corresponding to these detectors, $C=[c_{i,j}]$ is a 10×20 matrix with entries $c_{1,1}=c_{2,3}=c_{3,5}=c_{4,7}=c_{5,9}=c_{6,11}=c_{7,13}=c_{8,15}=c_{9,17}=c_{10,19}=1$ and $c_{i,j}=0$ for the other i,j. Moreover, one can verify that the pair $\left(A_{s},C\right)$ is observable for all s=1,⋯,20.

### 5.2. Analysis of Simulation Results

**Figure 8:**
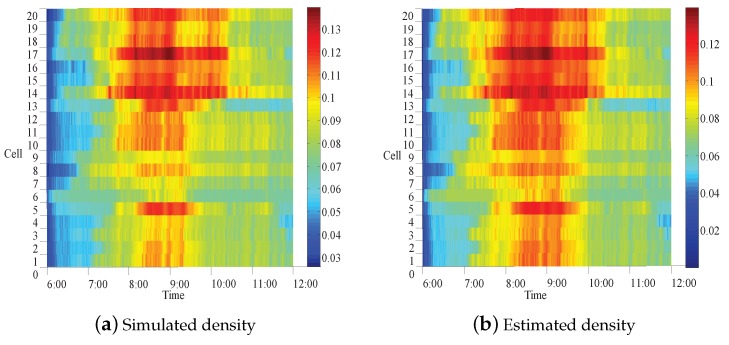
Time-space diagram of traffic density.

**Figure 9:**
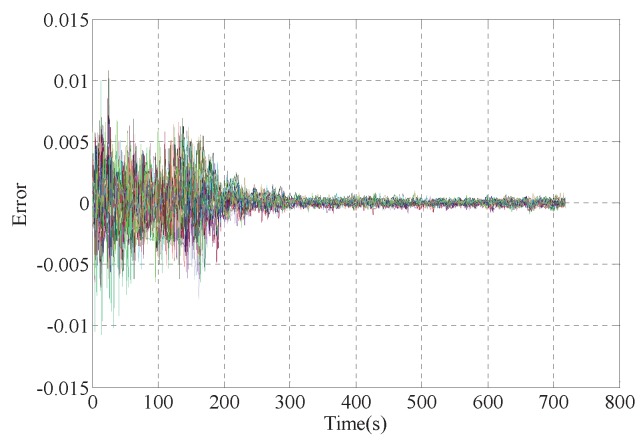
Error curve.

The simulation results for the whole simplified outer ring freeway are shown in Figure 8 in the way of time-space diagram, where Figure 8a shows the real traffic densities from 6:00 a.m. to 12:00 a.m., and Figure 8b presents the estimated densities by the observer. It is clear that some segments with red color represent a real congestion state. Especially, heavy traffic jams happen in the morning rush hour from about 7:00 a.m. to 10:00 a.m. and the congestion time periods are different for different road segments. Error curve(see Figure 9) shows that the error converges to zero at about 200 s, the speed is acceptable and the gain matrices are appropriate.

**Figure 10:**
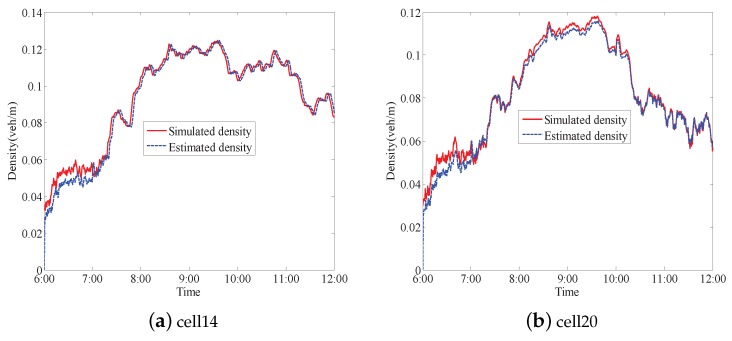
Simulated and estimated densities of cells 14 and 20.

One can further compare estimated densities with the simulated ones in all road segments. For example, the cells 14 and 20 are presented to exhibit more in detail the the density comparison (see Figure 10). From Figure 8 and Figure 10, it is clear that the proposed modeling approach and the observer are feasible, and the estimated densities well approximate to the simulated ones.

Table 4 shows the performance indicator of the state observer by using the mean square error (MSE) which is defined by the Equation (23)

(23) $$\begin{array}{c} \mathrm{MSE}=\sqrt{\frac{\sum_{i=1}^{n}{\left({\hat{\rho }}_{i}-\rho_{i}\right)}^{2}}{n}} \end{array}$$

From the MSE of each cell, we can further compute the mean value of 20 cells is approximately 11.34%. The results indicate that the designed model-based switched state observer has a good estimated performance.

## 6. Conclusions and Future Work

In the paper, a modeling framework named Dynamic Graph Hybrid Automata (DGHA) has been proposed by combining a dynamic graph with hybrid automata. Then this framework was applied to model traffic flow over an urban freeway network by embedding the Cell Transmission Model (CTM) into the DGHA.

In the modeling procedure, the dual digraph of road network structure was adopted to describe the road topology, hybrid automata were used to describe multi-modes of dynamic densities in road segments, and the nonlinear expressions of the transmitted traffic flow between two road segments were transformed into piecewise linear functions in terms of multi-mode switchings. By using a combination algorithm for the dynamics of traffic flow, the modeling procedure is modularized and rule-based, and thus is easily-extensible. Thus it can be used to model an urban freeway network with arbitrary topology structures and sizes. Furthermore, mode types and number in the model of the whole freeway network were analyzed, and, consequently, a Piecewise Affine Linear System (PWALS) model of urban freeway was deduced. Finally, based on the PWALS model, a switched state observer was designed to estimate the traffic densities of the urban freeway, where a set of observer gain matrices were computed by using the Lyapunov function approach. The proposed modeling approach and the observer were validated to be feasible by the simplified example of Beijing third ring freeway.

However, there are some drawbacks which require further improvements in the future research. First, we adopt centralized model and observer, which, as shown above, results in huge number of combined modes and computational difficulty. So from the view of practical application, decentralized model and decentralized observer are required to reduce computational complexity.

Second, the assumption that the switching function of the observer is the same with that of the model means that the switching of the observer is driven by the model states. However, in practice it may be difficult to implement and one needs to identify mode switchings by designing a discrete event observer, which will result in unsynchronized mode switchings between the practical object and the observer and thus bring about larger estimation error. So, in this case, another type of switched observer is required to be design for the state estimation.

Third, in the application example, we took the simplified version of Beijing third ring road, and used the incompletely collected traffic data and the simulation data obtained by Paramics. That means we need real experiments for the practical application to further verify our proposed approach.

## Figures and Tables

**Table 1:** Modes of *i* with relation to upstream *l*.

Mode	Condition of $\left(\rho_{l},\rho_{i}\right)$	Flow $q_{\mathrm{li}}$
FDF	$\begin{array}{c} 0\leq \rho_{l}\leq \alpha_{li},0\leq \rho_{i}\leq \rho_{i}^{0} \end{array}$	$V_{l}\rho_{l}$
FUF	$\begin{array}{c} C_{i}/V_{l}\leq \rho_{l}\leq \rho_{l}^{0},0\leq \rho_{i}\leq \rho_{i}^{0} \end{array}$	$C_{i}$
CDF/CUF	$\begin{array}{c} \rho_{l}^{0}\leq \rho_{l}\leq \rho_{l}^{m},0\leq \rho_{i}\leq \rho_{i}^{0} \end{array}$	$C_{l}\;\mathrm{or}\;C_{i}$
CDC	$\begin{array}{c} \rho_{l}^{0}\leq \rho_{l}\leq \rho_{l}^{m},\rho_{i}^{0}\leq \rho_{i}\leq \rho_{i}^{m}-C_{l}/W_{i} \end{array}$	$C_{l}$
CUC	$\begin{array}{c} \rho_{l}^{0}\leq \rho_{l}\leq \rho_{l}^{m},\beta_{li}\leq \rho_{i}\leq \rho_{i}^{m} \end{array}$	$W_{i}\left(\rho_{i}^{m}-\rho_{i}\right)$
FUC	$\begin{array}{c} 0\leq \rho_{l}\leq \rho_{l}^{0},\rho_{i}^{0}\leq \rho_{i}\leq \rho_{i}^{m},V_{l}\rho_{l}+W_{i}\rho_{i}\geq W_{i}\rho_{i}^{m} \end{array}$	$W_{i}\left(\rho_{i}^{m}-\rho_{i}\right)$
FDC	$\begin{array}{c} 0\leq \rho_{l}\leq \rho_{l}^{0},\rho_{i}^{0}\leq \rho_{l}\leq \rho_{i}^{m},V_{l}\rho_{l}+W_{i}\rho_{i}\leq W_{i}\rho_{i}^{m} \end{array}$	$V_{l}\rho_{l}$

**Table 2:** Cell length of simplified third ring freeway.

Number	Length	Number	Length	Number	Length
1	111 m	8	430 m	15	300 m
2	535 m	9	500 m	16	350 m
3	336 m	10	355 m	17	332 m
4	138 m	11	338 m	18	210 m
5	586 m	12	355 m	19	367 m
6	140 m	13	458 m	20	458 m
7	281 m	14	256 m		

**Table 3:** Road segments parameters.

Number	*V*	*W*	*C*	$\rho^{0}$	$\rho^{m}$
2,3,4	65	20	2800	46	185
7,8,9	63	21	2850	45	180
12,13,14	65	20	2760	44	182
17,18,19	62	19	2650	43	180
1,20	60	19	2450	40	170
5,6	58	18	2350	41	165
10,11	55	20	2200	40	150
15,16	50	19	2100	41	155

**Table 4:** Mean square error of estimated densities.

Number of cell	1	2	3	4	5	6	7	8	9	10
MSE	0.1095	0.1126	0.1132	0.1223	0.1205	0.1176	0.1301	0.1005	0.1108	0.1201
Number of cell	11	12	13	14	15	16	17	18	19	20
MSE	0.1132	0.1087	0.1136	0.1009	0.1143	0.1209	0.1120	0.1013	0.1147	0.1106

## Appendix A

$$H_{i1}=\left[\begin{array}{ccc} -1 & 0 & 0\\ 1 & 0 & 0\\ 0 & -1 & 0\\ 0 & 1 & 0\\ 0 & 0 & -1\\ 0 & 0 & 1 \end{array}\right],\;h_{i1}=\left[\begin{array}{c} 0\\ \rho_{l0}\\ 0\\ \rho_{i0}\\ 0\\ \rho_{j0} \end{array}\right]$$

$$H_{i2}=\left[\begin{array}{ccc} -1 & 0 & 0\\ 1 & 0 & 0\\ 0 & -1 & 0\\ 0 & 0 & -1\\ 0 & V_{i} & W_{j} \end{array}\right],\;h_{i2}=\left[\begin{array}{c} 0\\ \rho_{l0}\\ 0\\ -\rho_{j0}\\ W_{j}\rho_{jm} \end{array}\right]$$

$$H_{i3}=\left[\begin{array}{ccc} -1 & 0 & 0\\ 1 & 0 & 0\\ 0 & 1 & 0\\ 0 & 0 & 1\\ 0 & -V_{i} & -W_{j} \end{array}\right],\;h_{i3}=\left[\begin{array}{c} 0\\ \rho_{l0}\\ \rho_{i0}\\ \rho_{jm}\\ -W_{j}\rho_{jm} \end{array}\right]$$

$$H_{i4}=\left[\begin{array}{ccc} -1 & 0 & 0\\ 0 & -1 & 0\\ V_{l} & W_{i} & 0\\ 0 & 0 & -1\\ 0 & 0 & 1 \end{array}\right],\;h_{i4}=\left[\begin{array}{c} 0\\ -\rho_{i0}\\ W_{i}\rho_{im}\\ -\rho_{j0}\\ \rho_{jm} \end{array}\right]$$

$$H_{i5}=\left[\begin{array}{ccc} -1 & 0 & 0\\ 0 & -1 & 0\\ V_{l} & W_{i} & 0\\ 0 & 0 & -1\\ 0 & 0 & 1 \end{array}\right],\;h_{i5}=\left[\begin{array}{c} 0\\ -\rho_{i0}\\ W_{i}\rho_{im}\\ 0\\ \rho_{j0} \end{array}\right]$$

$$H_{i6}=\left[\begin{array}{ccc} -1 & 0 & 0\\ 1 & 0 & 0\\ 0 & -1 & 0\\ 0 & 1 & 0\\ 0 & 0 & -1\\ 0 & 0 & 1 \end{array}\right],\;h_{i6}=\left[\begin{array}{c} -\rho_{l0}\\ \rho_{lm}\\ 0\\ \rho_{i0}\\ 0\\ \rho_{j0} \end{array}\right]$$

$$H_{i7}=\left[\begin{array}{ccc} -1 & 0 & 0\\ 1 & 0 & 0\\ 0 & -1 & 0\\ 0 & 0 & -1\\ 0 & V_{i} & W_{j} \end{array}\right],\;h_{i7}=\left[\begin{array}{c} -\rho_{l0}\\ \rho_{lm}\\ 0\\ -\rho_{j0}\\ W_{j}\rho_{jm} \end{array}\right]$$

$$H_{i8}=\left[\begin{array}{ccc} -1 & 0 & 0\\ 1 & 0 & 0\\ 0 & 1 & 0\\ 0 & 0 & 1\\ 0 & -V_{i} & -W_{j} \end{array}\right],\;h_{i8}=\left[\begin{array}{c} -\rho_{l0}\\ \rho_{lm}\\ \rho_{i0}\\ \rho_{jm}\\ -W_{j}\rho_{jm} \end{array}\right]$$

$$H_{i9}=\left[\begin{array}{ccc} -1 & 0 & 0\\ 1 & 0 & 0\\ 0 & -1 & 0\\ 0 & 1 & 0\\ 0 & 0 & -1\\ 0 & 0 & 1 \end{array}\right],\;h_{i9}=\left[\begin{array}{c} -\rho_{l0}\\ \rho_{lm}\\ -\rho_{i0}\\ \rho_{im}\\ 0\\ \rho_{j0} \end{array}\right]$$

$$H_{i10}=\left[\begin{array}{ccc} 1 & 0 & 0\\ 0 & 1 & 0\\ -V_{l} & -W_{i} & 0\\ 0 & 0 & -1\\ 0 & 0 & 1 \end{array}\right],\;h_{i10}=\left[\begin{array}{c} \rho_{l0}\\ \rho_{im}\\ -W_{i}\rho_{im}\\ 0\\ \rho_{j0} \end{array}\right]$$

$$H_{i11}=\left[\begin{array}{ccc} 1 & 0 & 0\\ 0 & 1 & 0\\ -V_{l} & -W_{i} & 0\\ 0 & 0 & -1\\ 0 & 0 & 1 \end{array}\right],\;h_{i11}=\left[\begin{array}{c} \rho_{l0}\\ \rho_{im}\\ -W_{i}\rho_{im}\\ -\rho_{j0}\\ \rho_{jm} \end{array}\right]$$

$$H_{i12}=\left[\begin{array}{ccc} -1 & 0 & 0\\ 1 & 0 & 0\\ 0 & -1 & 0\\ 0 & 1 & 0\\ 0 & 0 & -1\\ 0 & 0 & 1 \end{array}\right],\;h_{i12}=\left[\begin{array}{c} -\rho_{l0}\\ \rho_{lm}\\ -\rho_{i0}\\ \rho_{im}\\ -\rho_{j0}\\ \rho_{jm} \end{array}\right]$$

## Appendix B

Mode 1: FFFFFFFFFFFFFFFFFFFF (20F)

$a_{1,1}=0.2491$, $a_{1,20}=0.7509$, $a_{2,1}=0.1558$, $a_{2,2}=0.8442$, $a_{3,2}=0.2481$, $a_{3,3}=0.7519$, $a_{4,3}=0.6040$, $a_{4,4}=0.3960$, $a_{5,4}=0.1422$, $a_{5,5}=0.8578$, $a_{6,5}=0.5954$, $a_{6,6}=0.4046$, $a_{7,6}=0.2966$, $a_{7,7}=0.7034$, $a_{8,7}=0.1938$, $a_{8,8}=0.8062$, $a_{9,8}=0.1667$, $a_{9,9}=0.8333$, $a_{10,9}=0.2348$, $a_{10,10}=0.7652$, $a_{11,10}=0.2466$, $a_{11,11}=0.7534$, $a_{12,11}=0.2348$, $a_{12,12}=0.7652$, $a_{13,12}=0.1820$, $a_{13,13}=0.8180$, $a_{14,13}=0.3256$, $a_{14,14}=0.6744$, $a_{15,14}=0.2778$, $a_{15,15}=0.7222$, $a_{16,15}=0.2381$, $a_{16,16}=0.7619$, $a_{17,16}=0.2511$, $a_{17,17}=0.7489$, $a_{18,17}=0.3969$, $a_{18,18}=0.6031$, $a_{19,18}=0.2271$, $a_{19,19}=0.7729$, $a_{20,19}=0.1820$, $a_{20,20}=0.8180$, and the other entries $a_{i,j}$ are zero. F=0.

Mode 2: FFFFFFFFFFFFFFFFCCCC (16F4C)

$a_{1,1}=0.2491$, $a_{2,1}=0.1558$, $a_{2,2}=0.8442$, $a_{3,2}=0.2481$, $a_{3,3}=0.7519$, $a_{4,3}=0.6040$, $a_{4,4}=0.3960$, $a_{5,4}=0.1422$, $a_{5,5}=0.8578$, $a_{6,5}=0.5954$, $a_{6,6}=0.4046$, $a_{7,6}=0.2966$, $a_{7,7}=0.7034$, $a_{8,7}=0.1938$, $a_{8,8}=0.8062$, $a_{9,8}=0.1667$, $a_{9,9}=0.8333$, $a_{10,9}=0.2348$, $a_{10,10}=0.7652$, $a_{11,10}=0.2466$, $a_{11,11}=0.7534$, $a_{12,11}=0.2348$, $a_{12,12}=0.7652$, $a_{13,12}=0.1820$, $a_{13,13}=0.8180$, $a_{14,13}=0.3256$, $a_{14,14}=0.6744$, $a_{15,14}=0.2778$, $a_{15,15}=0.7222$, $a_{16,15}=0.2381$, $a_{16,16}=0.7619$, $a_{17,16}=0.2511$, $a_{17,17}=1$, $a_{17,18}=0.0837$, $a_{18,18}=0.8676$, $a_{18,19}=0.1324$, $a_{19,19}=0.9243$, $a_{19,20}=0.0757$, $a_{20,20}=0.9363$, and the other entries $a_{i,j}$ are zero.

$f_{1}=0.0338$, $f_{17}=-0.0151$, $f_{20}=0.0027$, and the other entries $f_{i}$ are zero.

Mode 3: FFFFFFFFFFFFCCFFCCCC (12F2C2F4C)

$a_{1,1}=0.2491$, $a_{2,1}=0.1558$, $a_{2,2}=0.8442$, $a_{3,2}=0.2481$, $a_{3,3}=0.7519$, $a_{4,3}=0.6040$, $a_{4,4}=0.3960$, $a_{5,4}=0.1422$, $a_{5,5}=0.8578$, $a_{6,5}=0.5954$, $a_{6,6}=0.4046$, $a_{7,6}=0.2966$, $a_{7,7}=0.7034$, $a_{8,7}=0.1938$, $a_{8,8}=0.8062$, $a_{9,8}=0.1667$, $a_{9,9}=0.8333$, $a_{10,9}=0.2348$, $a_{10,10}=0.7652$, $a_{11,10}=0.2466$, $a_{11,11}=0.7534$, $a_{12,11}=0.2348$, $a_{12,12}=0.7652$, $a_{13,12}=0.1820$, $a_{13,13}=1$, $a_{13,14}=0.0607$, $a_{14,14}=0.8914$, $a_{15,15}=0.7222$, $a_{16,15}=0.2381$, $a_{16,16}=0.7619$, $a_{17,16}=0.2511$, $a_{17,17}=1$, $a_{17,18}=0.0837$, $a_{18,18}=0.8676$, $a_{18,19}=0.1324$, $a_{19,19}=0.9243$, $a_{19,20}=0.0757$, $a_{20,20}=0.9363$, and the other entries $a_{i,j}$ are zero.

$f_{1}=0.0338$, $f_{13}=-0.011$, $f_{14}=0.0049$, $f_{15}=0.0125$, $f_{17}=-0.0151$, $f_{20}=0.0027$, and the other entries $f_{i}$ are zero.

Mode 4: FFFFFFCCCFFFCCFFCCCC (6F3C3F2C2F4C)

$a_{1,1}=0.2491$, $a_{2,1}=0.1558$, $a_{2,2}=0.8442$, $a_{3,2}=0.2481$, $a_{3,3}=0.7519$, $a_{4,3}=0.6040$, $a_{4,4}=0.3960$, $a_{5,4}=0.1422$, $a_{5,5}=0.8578$, $a_{6,5}=0.5954$, $a_{6,6}=0.4046$, $a_{7,6}=0.2966$, $a_{7,7}=1$, $a_{7,8}=0.0989$, $a_{8,8}=0.9353$, $a_{8,9}=0.0647$, $a_{9,9}=0.9444$, $a_{10,10}=0.7652$, $a_{11,10}=0.2466$, $a_{11,11}=0.7534$, $a_{12,11}=0.2348$, $a_{12,12}=0.7652$, $a_{13,12}=0.1820$, $a_{13,13}=1$, $a_{13,14}=0.0607$, $a_{14,14}=0.8914$, $a_{15,15}=0.7222$, $a_{16,15}=0.2381$, $a_{16,16}=0.7619$, $a_{17,16}=0.2511$, $a_{17,17}=1$, $a_{17,18}=0.0837$, $a_{18,18}=0.8676$, $a_{18,19}=0.1324$, $a_{19,19}=0.9243$, $a_{19,20}=0.0757$, $a_{20,20}=0.9393$, and the other entries $a_{i,j}$ are zero.

$f_{1}=0.0338$, $f_{7}=-0.018$, $f_{9}=-0.0025$, $f_{10}=0.0106$, $f_{13}=-0.011$, $f_{14}=0.0049$, $f_{15}=0.0125$, $f_{17}=-0.0151$, $f_{20}=0.0027$, and the other entries $f_{i}$ are zero.

Mode 5: FFFFFFCCCFFFCCCCCCCC (6F3C3F8C)

$a_{1,1}=0.2491$, $a_{2,1}=0.1558$, $a_{2,2}=0.8442$, $a_{3,2}=0.2481$, $a_{3,3}=0.7519$, $a_{4,3}=0.6040$, $a_{4,4}=0.3960$, $a_{5,4}=0.1422$, $a_{5,5}=0.8578$, $a_{6,5}=0.5954$, $a_{6,6}=0.4046$, $a_{7,6}=0.2966$, $a_{7,7}=1$, $a_{7,8}=0.0989$, $a_{8,8}=0.9353$, $a_{8,9}=0.0647$, $a_{9,9}=0.9444$, $a_{10,10}=0.7652$, $a_{11,10}=0.2466$, $a_{11,11}=0.7534$, $a_{12,11}=0.2348$, $a_{12,12}=0.7652$, $a_{13,12}=0.1820$, $a_{13,13}=1$, $a_{13,14}=0.0607$, $a_{14,14}=0.8914$, $a_{14,15}=0.1086$, $a_{15,15}=0.9073$, $a_{15,16}=0.0927$, $a_{16,16}=0.9206$, $a_{16,16}=0.0794$, $a_{17,17}=0.9163$, $a_{17,18}=0.0837$, $a_{18,18}=0.8676$, $a_{18,19}=0.1324$, $a_{19,19}=0.9243$, $a_{19,20}=0.0757$, $a_{20,20}=0.9393$, and the other entries $a_{i,j}$ are zero.

$f_{1}=0.0338$, $f_{7}=-0.018$, $f_{9}=-0.0025$, $f_{10}=0.0106$, $f_{13}=-0.011$, $f_{20}=0.0027$, and the other entries $f_{i}$ are zero.

Mode 6: CCCCCCCCCCCCCCCCCCCC (16C)

$a_{1,1}=0.7495$, $a_{1,2}=0.2505$, $a_{2,2}=0.9480$, $a_{2,3}=0.0520$, $a_{3,3}=0.9173$, $a_{3,4}=0.0827$, $a_{4,4}=0.7986$, $a_{4,5}=0.2014$, $a_{5,5}=0.9526$, $a_{5,6}=0.0474$, $a_{6,6}=0.8014$, $a_{6,7}=0.1986$, $a_{7,7}=0.9011$, $a_{7,8}=0.0989$, $a_{8,8}=0.9353$, $a_{8,9}=0.0647$, $a_{9,9}=0.9444$, $a_{9,10}=0.0556$, $a_{10,10}=0.9217$, $a_{10,11}=0.0783$, $a_{11,11}=0.9178$, $a_{11,12}=0.0822$, $a_{12,12}=0.9217$, $a_{12,13}=0.0783$, $a_{13,13}=0.9393$, $a_{13,14}=0.0607$, $a_{14,14}=0.8914$, $a_{14,15}=0.1086$, $a_{15,15}=0.9073$, $a_{15,16}=0.0927$, $a_{16,16}=0.9206$, $a_{16,17}=0.0794$, $a_{17,17}=0.9163$, $a_{17,18}=0.0837$, $a_{18,18}=0.8676$, $a_{18,19}=0.1324$, $a_{19,19}=0.9243$, $a_{19,20}=0.0757$, $a_{20,20}=0.9393$, and the other entries $a_{i,j}$ are zero.

$f_{4}=0.7347$, $f_{6}=-0.8036$, $f_{9}=0.2167$, $f_{11}=-0.2630$, $f_{14}=0.3771$, $f_{16}=-0.1885$, $f_{19}=-0.0719$, and the other entries $f_{i}$ are zero.

Mode 7: FFFFFFFCFCCCFCCCFFFC

(7F1C1F3C1F3C3F1C)

$a_{1,1}=0.2491$, $a_{2,1}=0.1558$, $a_{2,2}=0.8442$, $a_{3,2}=0.2481$, $a_{3,3}=0.7519$, $a_{4,3}=0.6040$, $a_{4,4}=0.3960$, $a_{5,4}=0.1422$, $a_{5,5}=0.8578$, $a_{6,5}=0.5954$, $a_{6,6}=0.4046$, $a_{7,6}=0.2966$, $a_{7,7}=1$, $a_{7,8}=0.0989$, $a_{8,8}=0.9353$, $a_{8,9}=0.0647$, $a_{9,9}=1$, $a_{9,10}=0.0556$, $a_{10,10}=0.9217$, $a_{10,11}=0.0783$, $a_{11,11}=0.9178$, $a_{11,12}=0.0822$, $a_{12,12}=0.9217$, $a_{13,13}=1$, $a_{13,14}=0.0607$, $a_{14,14}=0.8914$, $a_{14,15}=0.1086$, $a_{15,15}=0.9073$, $a_{15,16}=0.0927$, $a_{16,16}=0.9206$, $a_{17,16}=0.9206$, $a_{17,17}=0.7489$, $a_{18,17}=0.3969$, $a_{18,18}=0.6031$, $a_{19,18}=0.2271$, $a_{19,19}=1$, $a_{19,20}=0.0757$, $a_{20,20}=0.9393$, and the other entries $a_{i,j}$ are zero.

$f_{1}=0.0338$, $f_{7}=-0.018$, $f_{8}=0.0029$, $f_{9}=-0.0025$, $f_{12}=0.0035$, $f_{12}=0.0027$, $f_{16}=0.0036$, $f_{17}=0.0113$, $f_{13}=0.0035$, $f_{19}=-0.0136$, $f_{20}=0.0027$, and the other entries $f_{i}$ are zero.
